# Integrated proteomic and transcriptomic landscape of human placenta in small for gestational age infants

**DOI:** 10.1016/j.isci.2024.111423

**Published:** 2024-11-19

**Authors:** Heyue Jin, Xianyan Wang, Lingyu Li, Chen Rui, Hong Gan, Qunan Wang, Fangbiao Tao, Yumin Zhu

**Affiliations:** 1 Department of Maternal & Child and Adolescent Health, School of Public Health, MOE Key Laboratory of Population Health Across Life Cycle, Anhui Provincial Key Laboratory of Population Health and Aristogenics, Anhui Medical University, Hefei, Anhui 230032, China; 2 Medical School, Nanjing University, Nanjing, Jiangsu 210093, China; 3 Department of Toxicology, School of Public Health, Anhui Medical University, Hefei, Anhui 230032, China

**Keywords:** Developmental anatomy, Pregnancy, Proteomics, Transcriptomics

## Abstract

Small for gestational age (SGA) infants affected by placental insufficiency are exposed to the risk of stillbirth and long-term complications. Based on RNA-seq and mass spectrometry, we identified dysregulated RNAs and proteins from the comparisons of SGA placental tissues and controls. We revealed two SGA-relevant co-expression modules (SRMs) that also significantly distinguished SGA from controls. Then we performed an integrated analysis of transcriptomic and proteomic profiles to trace their links to SGA as well as their significant correlations. For the core functional molecules we screened, we revealed their potential upstream regulators and validated them experimentally in an independent cohort. Overall, we pointed out insights into different molecular pathways for the pathological mechanisms of SGA and indicated potential target molecules that may be drivers of placental aberrations in the SGA infants.

## Highlights

• Proteomic and transcriptomic landscape of placenta were profiled
• Key regulatory genes for the small-for-gestational-age infants were depicted
• SGA-relevant co-expression modules were created
• The discussions of mRNA-protein correlations were expanded

## Graphical abstract

**Figure:**
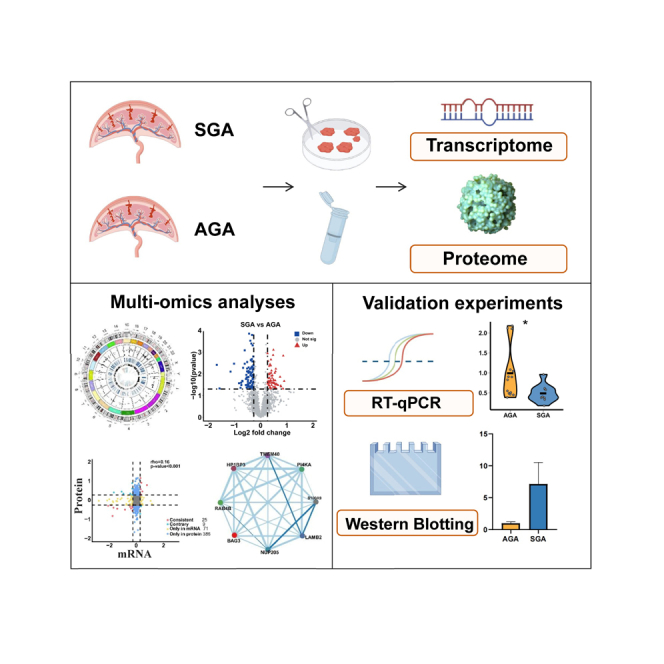

## Introduction

Small for gestational age (SGA) can be broadly defined as infants with birth weights below the 10 percentiles for that gestational age, accounting for about 16% of all births globally.^1^ SGA infants are associated with high perinatal morbidity and mortality, and this aberration in fetal growth may pose risks for health in later life, such as disorders of neurodevelopment, metabolic dysfunction, and cardiovascular diseases.^2^^,^^3^^,^^4^ In particular, SGA infants have a higher incidence of cognitive deficits that can lead to learning deficits.^5^ As a public health problem, SGA infants can also place a significant economic burden on their families and society. In total, the issue of the etiology of SGA deserves considerable attention in view of these risks.

Previous research has revealed that the causes of SGA comprise a multifactorial array, involving a combination of maternal health, placenta dysfunction, and genetic factors.^2^^,^^6^ Such complexity contributed to the etiological mechanisms that underlie SGA may remain understudied. The placenta serves as a critical window into the maternal-fetal interface, with its nutrient transfer capability being closely associated with fetal growth. Thus, the placenta is regarded as a key window for elucidating the potential or established pathological mechanisms underlying SGA and other adverse birth outcomes.^7^^,^^8^ Importantly, the relationship between the placenta and SGA can be elucidated through comprehensive placental gene expression profiling, made possible by advances in “omics” technologies and the decreasing cost of sequencing.^9^^,^^10^ Several studies have sought to explain the pathogenesis of SGA by analyzing transcriptomic changes through human placental bulk mRNA and miRNA sequencing of SGA infants.^8^^,^^11^ Although most efforts to date have focused on measuring RNA, providing a valuable resource for uncovering the biological mechanisms underlying SGA, there are limitations in using single omics approaches, such as transcriptomics, to fully reveal SGA pathogenesis. The ultimate biological effectors of diseases are often the proteins it modulates,^12^ given proteins reside downstream of transcription and involve more directly in important cellular activities, proteome is expected to complement transcriptome and furnish an understanding of post-transcriptional regulatory mechanisms, and molecular mechanisms in SGA.^13^^,^^14^ In particular, to our knowledge, none of the previous studies has used transcriptomic profiles and corresponding proteomic information in the placenta to systematically portray molecular explanations for the occurrence of SGA.

To this end, we hypothesized that proteo-transcriptomic consolidation would reveal pivotal disease features beyond a single technology, and applied integrated analyses of the placenta proteomic and transcriptomic data for 62 pregnancies, to investigate the potential SGA-associated regulatory genes. In addition, we characterized the pertinence between protein levels and transcript levels in the placenta tissues, along with the correlation between protein levels and transcript levels between SGA and average for gestational age (AGA) infants. Our findings unveil the aberrant molecules expression in the placenta with SGA and deliver a valuable resource to better understand the relationships among multi-omics data in the context of SGA.

## Results

### RNA profiling across placentas from SGA and AGA groups

**Figure 1:**
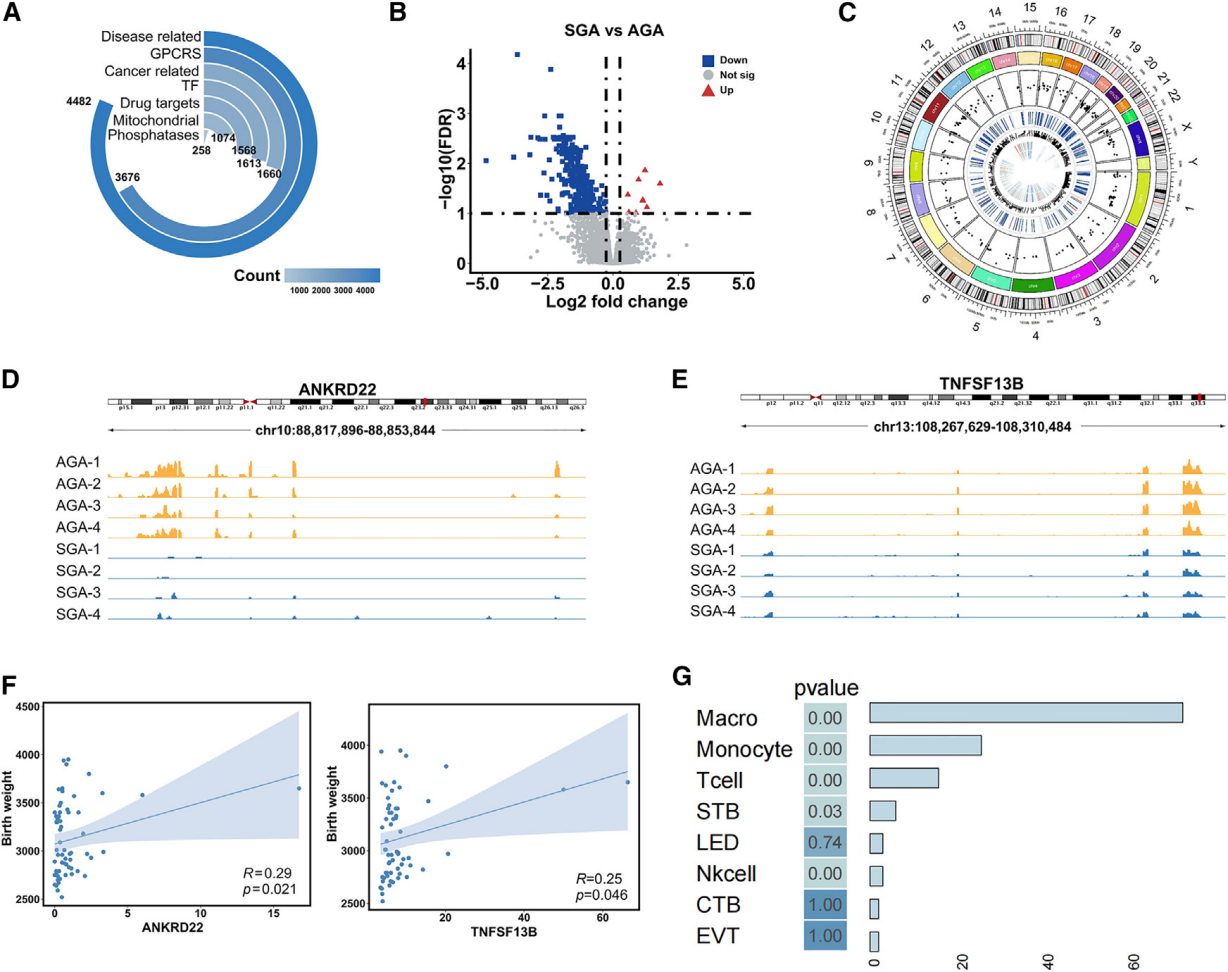
The transcriptomic characteristics of SGA (A) Distribution of detected RNAs in selected functional categories. (B) Volcano plots of DEGs in the comparison of SGA and AGA groups. (C) Visualization of DEGs chromosome positions in a circos plot. The innermost circle is a heatmap of the average expression levels of DEGs in the SGA and AGA groups, the second circle is the expression trend of DEGs, with red denoting upregulated and blue denoting downregulated, and the third circle is the log2 fold change of DEGs, with each dot representing a gene. (D) Examples of *ANKRD22* in expression patterns. (E) Examples of *TNFSF13B* in expression patterns. (F) The correlation between birth weight and the expression levels of *ANKRD22* and *TNFSF13B*. (G) Assessment of the cell specificity of DEGs. The bars represent the number of genes where DEGs overlaps with the set of cellular marker gene sets.

For this study, we analyzed a total of 62 placenta tissues of SGA and AGA infants (*n* = 31 SGA, *n* = 31 AGA) from the Ma’ anshan birth cohort (MABC) study by RNA-seq based transcriptomic (Table S1). Pregnant women in the SGA group delivered at 39.6 ± 0.5 weeks (mean ± SD), this group consisted of 15 female placentas and 16 male placentas, and the fetal birth weight was 2807.1 ± 126.8 g (mean ± SD). In the AGA group, 31 women delivered at 39.5 ± 0.5 weeks (mean ± SD), this group consisted of 16 female placentas and 15 male placentas, and the fetal birth weight was 3432.9 ± 265.9 g (mean ± SD). Then we ultimately identified 60,065 total RNAs from these samples, ranging from 29,000 to 37,000 in each sample (Figure S1A). Subsequently, we found that most of the genes detected in the placenta are disease-related, and this distribution trend is the same as the overall trend detected in 29 human tissues (Figure 1A).^15^ We have detected several genes in placental tissues that perform drug target actions, in particular G-protein-coupled receptors (GPCRs), the expression of which has been known to vary considerably by tissue (Figure 1A).^16^ We showed that the number of genes associated with cancer and transcription factors (TFs) is nearly equivalent, while mitochondrial genes and phosphatases were found in a narrow quantity in the placenta (Figure 1A). This probably demonstrates the functional specialization of RNAs expressed in the placenta. To investigate the potential roles of RNAs in SGA, we identified a total of 342 differentially expressed genes (DEGs) when comparing all 31 SGA placentas with 31 matched AGA placentas. Among these, 9 were up-regulated and 333 were down-regulated (Figure 1B). A significant proportion of the 342 DEGs were mRNAs (82.2%), with other types including ncRNAs (7.3%), lncRNAs (6.1%), and pseudogenes (4.4%) (Figure S1B). This distribution contrasts with the overall functional representation of the total genes, which were largely dominated by GPCRs in the set of DEGs (Figure S1C).

We visualized the chromosomal locations of all DEGs, along with their log2 fold change (FC) values and expression patterns. These DEGs are distributed across all chromosomes except the Y chromosome, with the highest concentrations found on chromosomes 19, 1, and 11 (Figure 1C). To evaluate the functional relevance of our datasets, we compared our DEGs with reliable SGA-associated genes identified from previous literature.^8^ For the overlapping DEGs with reported SGA-associated genes, we extracted two representative genes with pronounced differential expression trends between the SGA and AGA groups in the peak plot. Interestingly, our findings also manifest that the transcriptomic expression pattern of the placenta from the SGA case is tangibly distinct from the corresponding control (Figures 1D and 1E). Compared to the AGA group, the expression levels of *ANKRD22* and *TNFSF13B* are lower in the SGA group (Figures S1D and S1E). Furthermore, in the correlation analysis, we captured a significantly positive relationship between the expression level of the two genes (*ANKRD22*, *TNFSF13B*) and birthweight (R ≥ 0.25, *p* value<0.05) (Figure 1F). According to previous studies, *TNFSF13B* is a well-documented B cell activating factor that, when expressed in the placenta, may promote the maturation, development, and differentiation of placental cells. Additionally, it plays a role in the maternal immune system during pregnancy, potentially affecting fetal growth in the womb.^17^^,^^18^^,^^19^ We proposed the evidence for the connection of *TNFSF13B* and regarding development in the placenta based on our data. In this context, these findings suggest that RNAs may serve as core regulatory molecules in SGA, establishing a foundation for further exploration.

By comparing the DEGs with cell marker genes presented in published studies, we also assessed aberrant regulatory genes in the SGA placenta and whether were enriched in cell-type-specific signatures. Our DEGs showed strong enrichment in macrophages, monocytes, T cells, syncytiotrophoblast (STB), and natural killer (Nk) cells (*p* value<0.05) (Figure 1G). We particularly noted the highest number of DEGs enriched in macrophages, which are a major source of pro-inflammatory chemokines and have the capacity to secrete a wide range of cytokines for inflammatory responses and mediate immunosuppression.^20^^,^^21^ The subpopulation of Nk cells present early in pregnancy has also been reported to influence fetal development and birth weight by secreting growth-promoting factors.^22^ Collectively, this may suggest dysregulation of the immune system plays a crucial role in the pathogenesis of SGA.

### Identification of SGA-relevant co-expression modules

**Figure 2:**
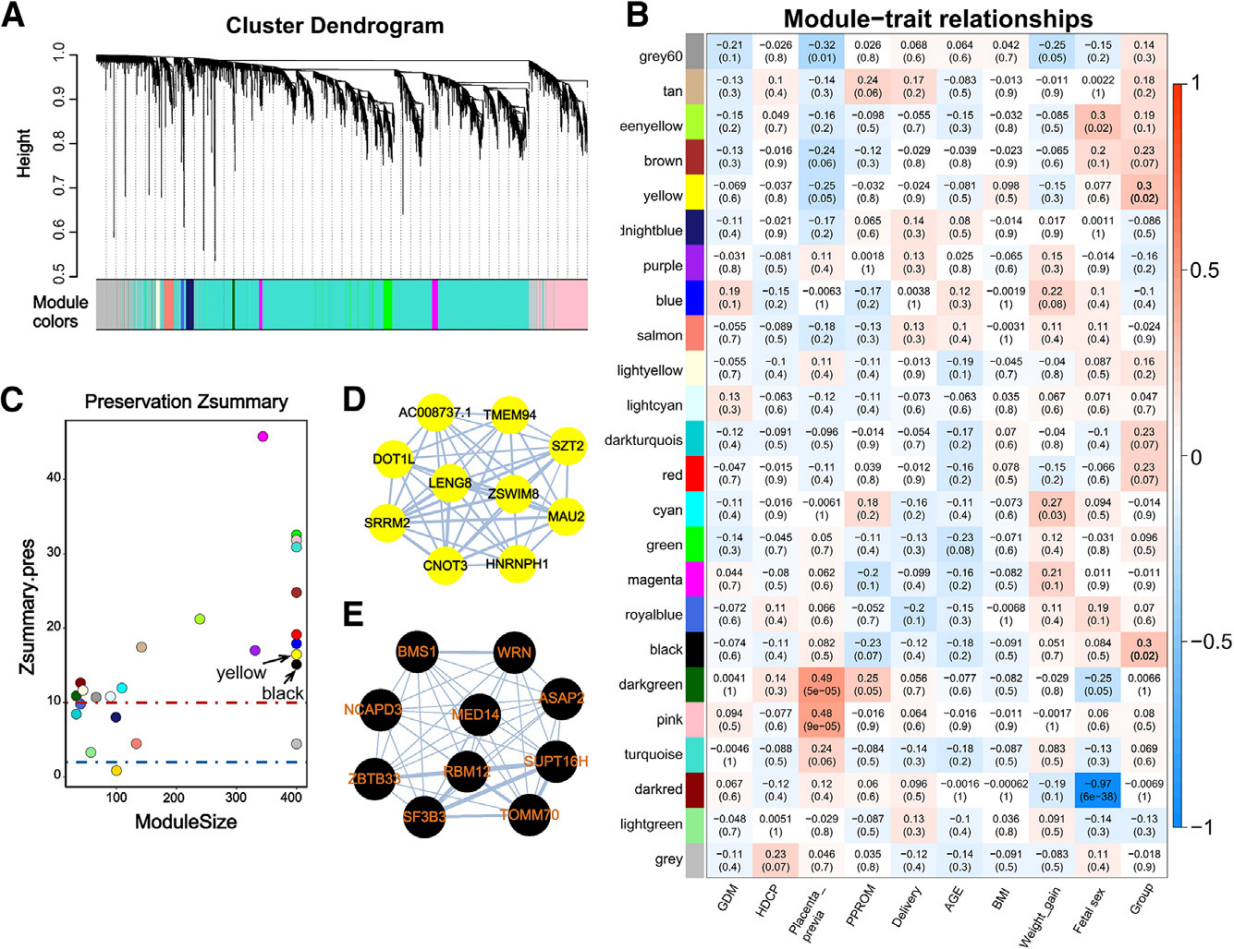
The gene co-expression networks and SRMs (A) The cluster of modules. (B) Pearson correlation analysis of network modules with clinical characteristics. The modules framed by the red dotted line are the ones with the strongest correlation and significance with SGA. GDM, gestational diabetes mellitus (any level of the early or initial diagnosis of glucose intolerance in pregnancy). HDCP, hypertensive disorder complicating pregnancy. PPROM, preterm premature rupture of membranes. (C) Calculation of Module preservation. The red dashed line (*Z* score = 10) represents the threshold line, above which considered be preserved. (D and E) Top 10 gene with the highest connectivity in the SRMs.

To clarify if the gene co-expression network works in SGA, we used 23,115 RNAs after filtering to build a gene co-expression network by the weighted correlation network analysis (WGCNA) algorithm (see methods), and clustered the transcriptomic profiles of SGA and AGA (Figure 2A). We identified a total of 24 modules and calculated the relationship between modules based on eigengenes from modules, then we found strong correlations between these modules internally and mostly positive (Figure S2A). Numbers of genes in each module are unequal and the turquoise module contained the highest number of genes (Figure S2B). Two of the 24 modules (yellow and black modules) show a significantly positive correlation between the SGA cases and controls (R = 0.3, *p* value = 0.02) (Figure 2B). Further assessment of the module preservation determined that the yellow module and black module can be preserved in the RNA network (Figure 2C). Therefore, we establish these two module networks as SGA-related network modules, defined as “SRM” (Figures 2D and 2E). Pathway analyses based on SRMs revealed that they both enriched in the term of regulation of the cellular metabolic process, respectively, enriched in the metabolic process and biosynthetic process (Figure S2C). Characterized genes constructed into SRMs both showed significant enrichment in the cytotrophoblasts (CTB) and lymphatic endothelial decidual (LED) cell-specific gene sets (*p* value<0.05) (Figure S2D). Together, we thus identified two prominent SRMs through the co-expression network that enhanced the credibility that metabolism abnormalities work in the etiology of SGA.

### Protein profiling across placentas from SGA and AGA groups

**Figure 3:**
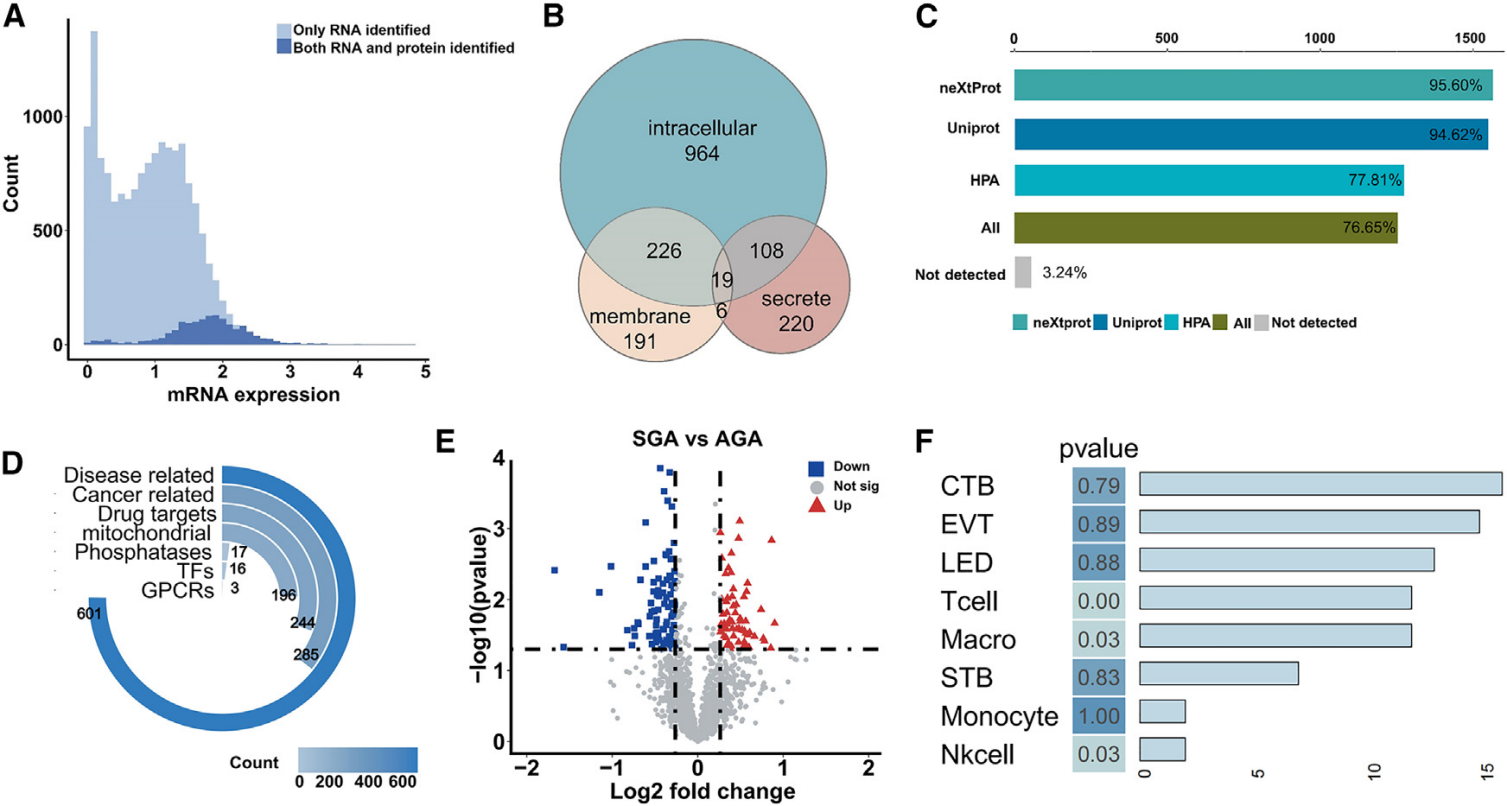
The proteomic characteristics of SGA (A) Distribution of detected proteins at the corresponding mRNA level. (B) The number of genes in each proteomic category: membrane, secreted, and intracellular. (C) The proportion of genes with access to protein evidence and not available in any of the three databases. “All” means that protein evidence exists in all three databases. (D) Distribution of detected proteins in selected functional categories. (E) Volcano plots of DEPs in the comparison of SGA and AGA groups. (F) Assessment of the cell specificity of DEPs. The bars represent the number of genes where DEPs overlap with the set of cellular marker gene sets.

We next profiled the proteome of the same samples that were employed for RNA-seq and detected proteins encoded by 1,625 genes across all placenta tissues. The overall range of mRNA expression encompassed a large proportion of the detected proteins, with identified proteins concentrated in regions of high mRNA levels (Figure 3A). In contrast, low-abundance mRNAs were scarcely detected at the protein level (Figure 3A).^14^ All detected proteins have been assigned according to protein isoforms including intracellular, membrane, and/or secreted.^23^ Notably, a larger proportion of intracellular proteins were detected compared to membrane and/or secreted proteins (Figure 3B). We have examined the number of proteins for which acquired protein evidence by combining verified antibody-based data with summary annotations performed by the Human Protein Atlas (HPA), UniProt, and neXtProt consortia. In our study, 95.9% of the detected proteins were identifiable from at least two efforts and 77.81% of proteins were identifiable from at least three efforts (Figure 3C). Only 3.24% of proteins lacked experimental evidence in currently available resources, though some may be identified in future updates (Figure 3C). We similarly attempted to characterize the potentially relevant functions of the detected proteins. Consistent with the distribution of RNA, we observed an extensive range of functionally categorized proteins linked to disease and cancer (Figure 3D). On the contrary, GPCRs represent the largest family of human membrane proteins, however, underrepresented in the distribution of all detected proteins may be due to the inadequate number of membrane proteins detected in our study (Figure 3D).

In the comparison of all SGA and AGA placentas, we found 157 proteins with differential expression, including 60 proteins with elevated expression and 97 proteins with reduced expression (Figure 3E). In keeping with the overall trend, most differentially expressed proteins (DEPs) continued to be disease-related, suggesting again the role of proteins in transmitting information to phenotypes associated with disease (Figures S3A and S3B).^24^ We also examined DEP enrichment in cell-type-specific gene sets. This analysis exhibits the enrichment of DEPs among macrophages, T cells, and STB gene sets, however, differences without the enrichment for the gene sets of Nk cells and monocytes compared to the results of DEGs (Figure 3F). This discrepancy may be due to the fact that the two molecules are differently controlled by dynamic processes including cell differentiation and stress states.^14^

### Pathways and sex patterns associated with SGA

**Figure 4:**
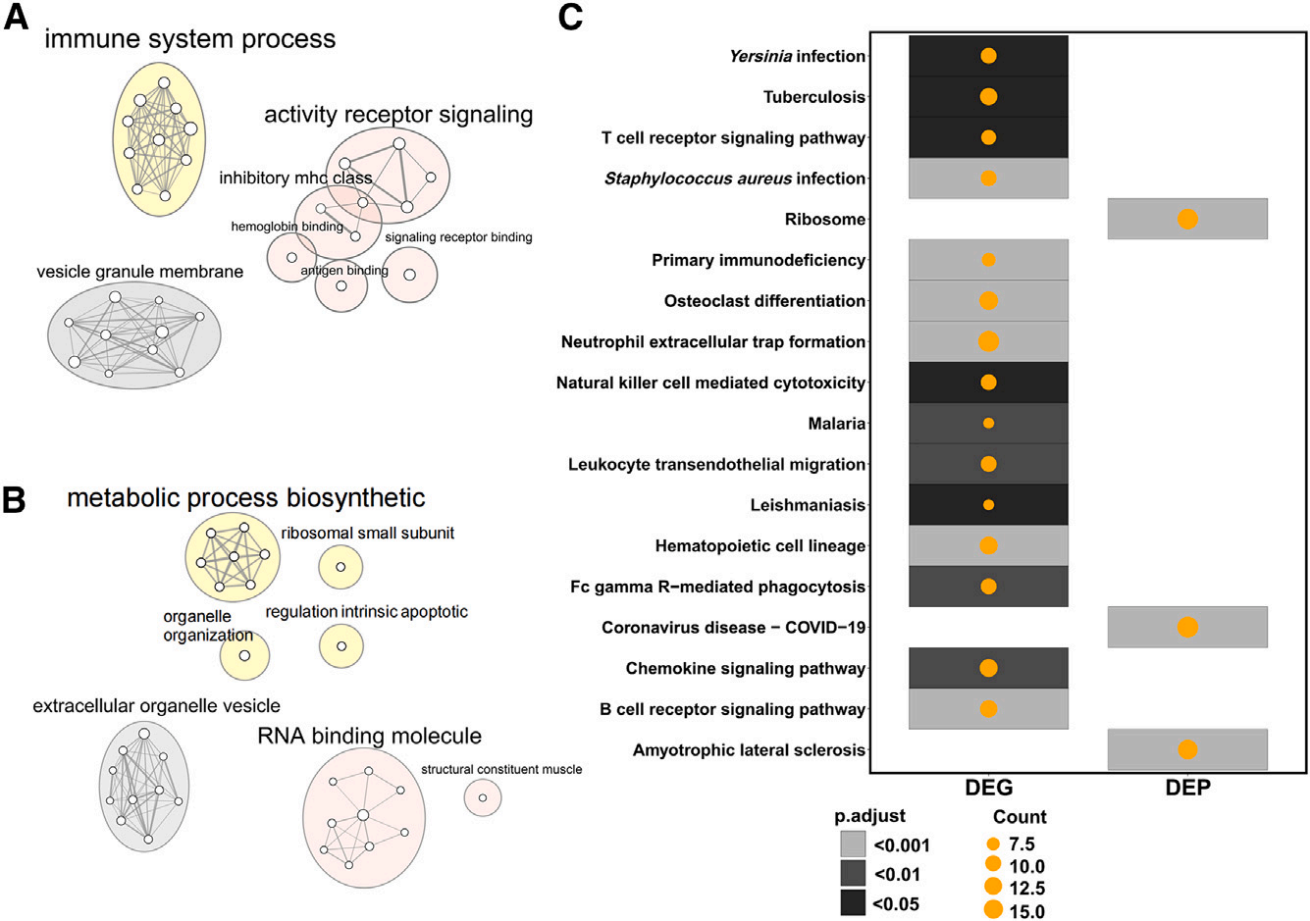
Pathways enriched for DEGs and DEPs (A) The top 10 GO-terms enriched in the DEGs. (B) The top 10 GO-terms enriched in the DEPs. Yellow represents the cluster of BP terms. Pink represents the cluster of MF terms. Gray represents the cluster of CC terms. The top term filtered according to adjusted *p* value by two-sided Fisher’s exact test. The pathway names on the graph are summaries of functionally similar pathways that form clusters, and the font size is derived from the word frequency of the pathway names. (C) The KEGG pathways enriched in the DEGs and DEPs.

To examine whether RNAs and proteins with altered expression in the SGA placenta were involved in different functional pathways, we further performed pathway enrichment analyses for DEGs and DEPs separately. We observed that the DEGs mainly function as an immune system process in biological process (BP) terms (Figure 4A). Proteins that were significantly altered in SGA clustered in distinct BP commonly related to metabolic process biosynthetic, ribosomal small subunit, organelle organization, and regulation of intrinsic apoptosis (Figure 4B). The enrichment of molecular function (MF) term showed that these DEGs focus on activity receptor signaling, inhibition mhc class, and receptor binding, while the DEPs engaged in RNA binding molecule and structural constituent muscle (Figures 4A and 4B). In cellular component (CC) terms, DEGs were predominantly associated with the vesicle granule membrane, whereas DEPs were linked to extracellular organelle vesicles (Figures 4A and 4B).

Moreover, there are marked differences in the enriched Kyoto Encyclopedia of Genes and Genomes (KEGG) pathways for DEGs and DEPs. We observed the DEGs enriched in the signal transduction pathways, immune related, and virus infection pathways, while the DEPs only enriched in ribosome, coronavirus disease-COVID-19, and amyotrophic lateral sclerosis pathways (Figure 4C). No shared KEGG pathways were identified between the two groups.

In addition to the global pathway analyses aforementioned, we conducted specific analyses for each sex group. We observed 27 DEGs between SGA and controls in males (10 up-regulated, 17 down-regulated) and 337 DEGs (13 up-regulated, 324 down-regulated) in females, indicating a distinctly skewed distribution (Figure S4A). At protein levels, we identified 98 DEPs between SGA and controls in males (46 up-regulated, 52 down-regulated) and 127 DEPs (64 up-regulated, 63 down-regulated) in females (Figure S4A). The down-regulated genes identified in the female group largely overlap with those identified in the sex-independent analysis, showing a lack of specificity. In contrast, the male group exhibits a higher number of DEG and DEP with distinct specificity (Figure S4B). Female-specific pathways of DEGs from the female placenta are mainly linked to the immune response, while no pathway was also detected to significantly enrich DEGs from male placenta (adjusted *p* value>0.05), which may be related to the lower number of male-specific DEGs (Figure S4C). We also found female-specific DEPs were enriched in pathways related to translation, extracellular matrix composition, and non-integrin membrane-ECM interactions, which may suggest a potential mechanism by which the binding of transmembrane proteoglycans to growth factor receptors influences cellular functions (Figure S4C). Male-specific DEPs were related to mRNA processing, the absence of Hedgehog signaling, and differentiation of hematopoietic stem cells (Figure S4C). The previous results may imply that the total DEGs and DEPs have different underlying mechanisms in the regulation of SGA, and specially revealed the sex-specific pathways by sub-sex analyses.

### Combination analysis of mRNA-level and protein-level data

**Figure 5:**
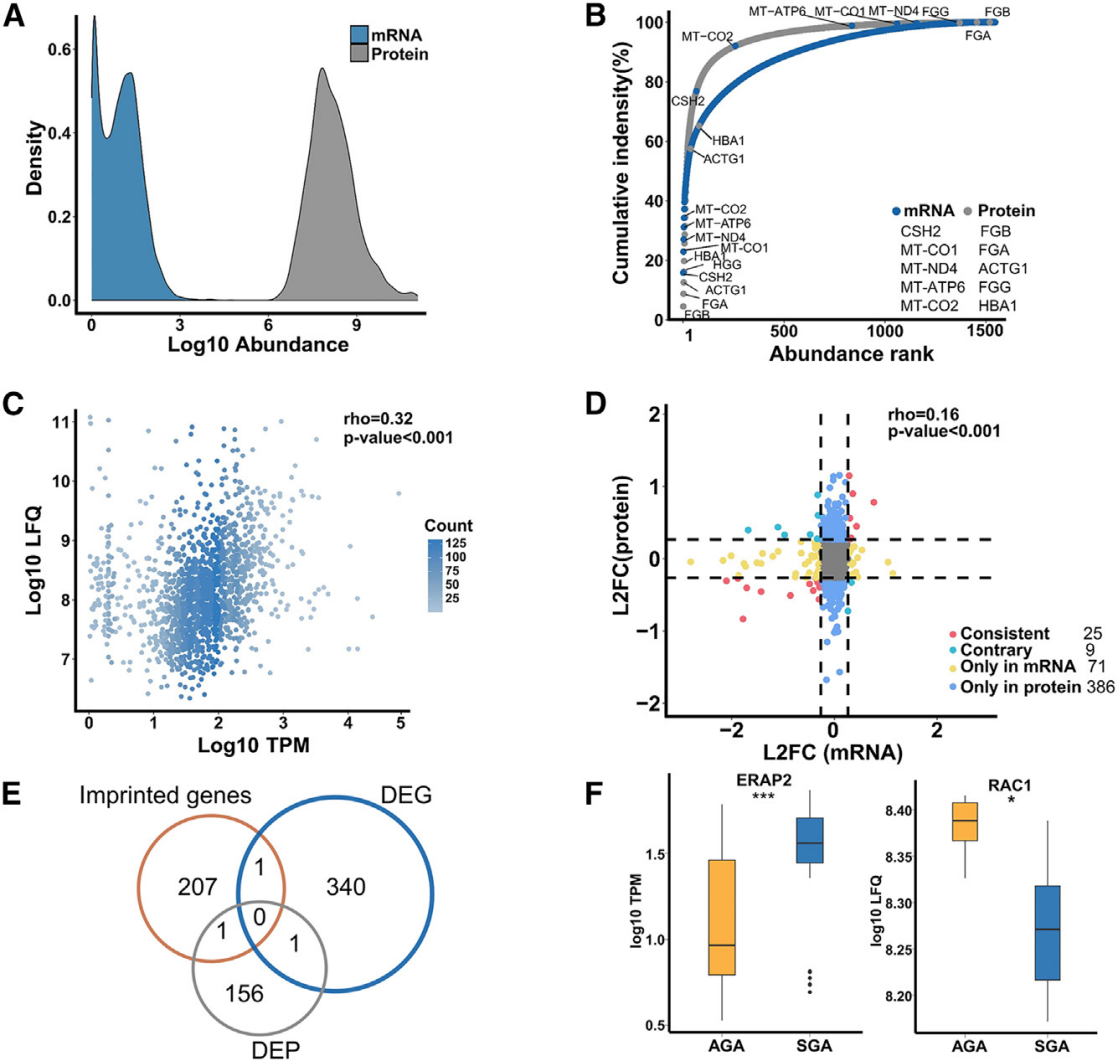
Combined analysis of mRNA and protein expression (A) The dynamic range of mRNA and protein expression. (B) Ranked abundance plot of mRNAs and proteins. The top five sorted mRNAs and proteins are labeled. (C) Scatterplot of correlation between mRNA and protein expression. (D) Five-quadrant plot of mRNA and protein expression patterns. L2FC, log2 fold change. (E) Venn diagram shows overlapped imprinted genes. (F) The comparison of expression levels of imprinted genes *ERAP2* and *RAC1* in the SGA and AGA groups. ∗*p* value<0.05, ∗∗∗*p* value<0.001. Boxes represent the interquartile range (IQR) with lines showing the median and whiskers denoting the smallest and largest values within 1.5∗IQR. The *p* value was calculated by Mann-Whitney test.

The relationship between protein abundance and mRNA expression has long been a subject of intense and ongoing debate.^25^^,^^26^^,^^27^ In an effort to shed light on this relationship, we aimed to provide a resource from placental tissue that profiles the association between protein and RNA, with the goal of ultimately clarifying their interaction, despite the current lack of consensus. We systematically described the distribution of mRNA-level and protein-level abundance based on measurements generated by different detection techniques. The protein abundance covered a dynamic range of roughly five orders of magnitude while the concentrations of mRNA spanned nearly five orders of magnitude but slightly less than that of protein (Figure 5A). Compared to previous observations in 29 human tissues,^15^ the difference observed in our study appears to be less pronounced, likely due to the limited detection of low-abundance protein molecules. Given the limitations of mass spectrometry in detecting low-abundance molecules in our study, this may explain the discrepancy we observed between the overall coverage of expressed genes acquired by RNA-seq and the coverage of protein abundance by liquid chromatography-mass spectrometry (LC-MS). We computed the rank order of the mRNA and protein abundance to further reveal the difference in their expression. We found that approximately 34% of the total mRNA abundance was contributed by top five mRNAs (*CSH2*, a single mRNA represents 15%), however, the top five proteins in abundance order, merely represent nearly 20% of the total protein intensity (Figure 5B). Interestingly, the previous top five mRNAs ranked low in abundance at the protein level, and a similar pattern was observed in the ranking of the top five proteins at the corresponding mRNA level (Figure 5B). The previous results reflected mRNA and protein abundance generally shows an uneven distribution.

The analysis of correlations between the mRNA and protein is typically necessary to clarify the relationship between abundance at transcriptional and translational level. We also unraveled the positive correlation between them in human placenta tissue based on the rho calculated by Spearman correlation is 0.32 (Figure 5C). Meanwhile, we captured the significance of this correlation based on the reported *p* value (*p* value<0.001).

We depicted a scatterplot containing log2 FC ratios at mRNA and protein levels to shed light on the regulatory mechanisms of gene expression with mRNA and protein correlations for SGA. Based on the alterations in the expression patterns of mRNA and protein between the SGA and AGA groups, we categorized the genes into five quadrants: consistent (consistent regulatory pattern at mRNA and protein levels), contrary (discordant regulatory pattern at mRNA and protein levels), only in mRNA (only differentially expressed at mRNA levels, with no change at protein levels), only in protein (only differentially expressed at protein levels, with no change in mRNA levels), and not (no change at both mRNA and protein levels). As a whole, the alterations at mRNA and protein levels between SGA and AGA groups remained significantly correlated (*p* value<0.001, Figure 5D). In our observation, it is evident that regulation at the translational level leads to the formation of a gene set labeled “Only in protein”, which encompasses the largest number of genes (Figure 5D). This may suggest a crucial role for proteins in the SGA regulatory mechanism. Additionally, we observed a small group of genes displaying opposite variations at the mRNA and protein levels (the contrary group), indicating a possible post-transcriptional regulatory mechanism for SGA. Excluding the gene set with no alterations at either mRNA or protein levels, we conducted pathway enrichment analyses on the remaining four gene sets. These analyses revealed a significant enrichment in the cytoplasmic translation term (Figure S5A). Three gene sets (“Consistent”, “Only in mRNA”, and “Only in protein”) were significantly enriched in the extracellular pathways of the CC terms containing extracellular vesicle, extracellular membrane-bounded organelle, extracellular exosome, and extracellular organelle (Figure S5A), which be related to the fact that the placenta is an important source of circulating exosomes, which release exosomes and other extracellular vesicles capable of mediating interactions between maternal and fetal cells during pregnancy (Figure S5A).^28^^,^^29^ In addition, the “Only in protein” set showed significant enrichment in MF terms cell as protein binding, cadherin binding, and cell adhesion molecule binding, while the “Only in mRNA” set prominently involved in MF terms of structural molecule activity and “Consistent” gene set involved in haptoglobin binding and oxygen carrier activity (Figure S5A). The previous shows that the crucial molecular activities and the binding of different glycoproteins contribute to the understanding of SGA pathogenesis. Regarding the KEGG pathways, we perceived several genes related to the infectious diseases, such as amebiasis and COVID-19. As indicated in prior reports,^30^^,^^31^ several bacterial, viral, and parasitic infections can interfere with fetal growth through placental inflammation, alternatively, these pathogens can penetrate placental defense mechanisms and cause fetal infections through vertical transmission at the mother-fetal interface, which can result in adverse outcomes. Therefore, building on previously reported mechanisms associated with SGA,^2^ we further systematically investigated whether these genes could be linked to SGA-associated diseases including maternal infectious diseases (malaria, Zika virus), fetal genetic disorders (Silver-Russell syndrome, temple syndrome), disorders of the growth hormone (GH) -IGF axis (GH deficiency, Laron syndrome), and disorders of affecting paracrine factors (hypochondroplasia, achondroplasia). Most of these genes were found in disease-associated datasets e.g., malaria, GH deficiency, and temple syndrome (Figure S5B). For instance, placental inflammation and subsequent impaired placental growth are hypothesized to be involved in the mechanism of malaria-associated birth weight loss.^32^^,^^33^ GH deficiency is known to be associated with decreased mean birth weight and length, and GH therapy has been shown to promote growth in children born with SGA and persistent short stature.^2^ Overall, our studies of SGA regulatory molecules provide valuable insights into the disease mechanisms and treatment of SGA. Apart from this, previous evidence has demonstrated that placental imprinted genes function indispensably in fetal growth and development.^34^^,^^35^^,^^36^ Here, we identified one DEG (*ERAP2*) and DEP (*RAC1*) that overlap with the list of imprinted genes integrated by searching the Geneimprint database and published literature, respectively (Figures 5E and 5F).^37^^,^^38^^,^^39^^,^^40^

### Identification of key regulatory molecules

**Figure 6:**
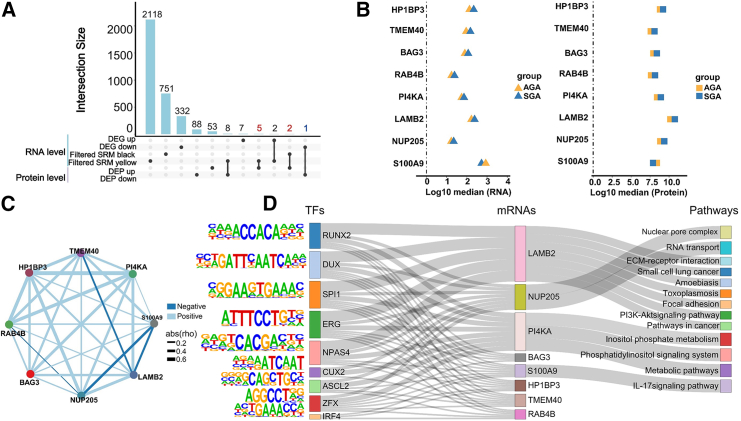
Identification of key regulatory molecules of SGA (A) The upset plot of genes associated with SGA at the RNA and protein level. Red represents genes that are coherently upregulated at the transcriptomic and proteomic levels, and blue represents genes that are coherently downregulated. (B) The comparison of the median value at RNA and protein levels of eight regulatory genes. The black dotted line represents a median value of 1. (C) Network diagram of correlations between genes. (D) Sankey diagram demonstrates the relationship between TFs and their target mRNAs, and the KEGG pathway enriched for target mRNAs.

Furthermore, we sought to identify a set of key regulatory molecules related to SGA from the results of the transcriptomic and proteomic analyses. Initially, we filtered all genes in the two positively correlated SRMs identified in WGCNA based on the condition that the median expression level of each gene in the SRMs of the SGA group exceeded the median level of the AGA group. As previously described, SRMs are modules that are positively correlated with the SGA grouping. Thus, we overlapped SRM genes with upregulated RNAs and upregulated proteins, and similarly overlapped downregulated RNAs with downregulated proteins to identify genes with consistent regulatory trends at both the transcriptional and translational levels. Next, we overlapped the DEGs, the filtered SRMs, and DEPs (Figure S6). We identified seven genes that are overexpressed in the SGA group at both the RNA level and the protein level and one shared downregulated gene at the RNA level and the protein level (Figure 6A). Immediately, we examined whether the median value of these genes in the low-expression group was more significant than 1. For example, the median level of the downregulated gene *S100A9* in the SGA group was greater than 1 (Figure 6B). All eight genes met these criteria at both the RNA level and the protein level (Figure 6B). Consequently, we identified eight genes as SGA-related key regulatory molecules for subsequent analysis and validation. We measured the correlation among these eight genes, indicating a positive correlation between these genes (Figure 6C). TFs are key elements in the regulation of gene expression. To explore whether these core regulatory molecules are regulated by specific TFs, we used HOMER to predict binding TFs and corresponding sequence motifs, which facilitate the construction of the TF-mRNA network (Table S3). Nine TFs such as *DUX*, *SPI1*, and *ERG* targeted the total eight genes, and may collaboratively regulate the downstream pathways of metabolic and virus infection, which are the pivotal paths in the SGA (Figure 6D). Notably, *ERG* has been identified as a differentially methylated gene relevant to fetal growth.^41^ Many fetuses labeled as SGA at birth can be diagnosed with skeletal dysplasia after birth.^2^ Coincidentally, *RUNX2* has been implicated in perinatal chondrocyte formation, contributing to postnatal bone development.^42^ These observations suggest that several TFs may be synergistically involved in the dysregulation of the SGA placenta.

### Validation of key regulatory molecules in the placenta

**Figure 7:**
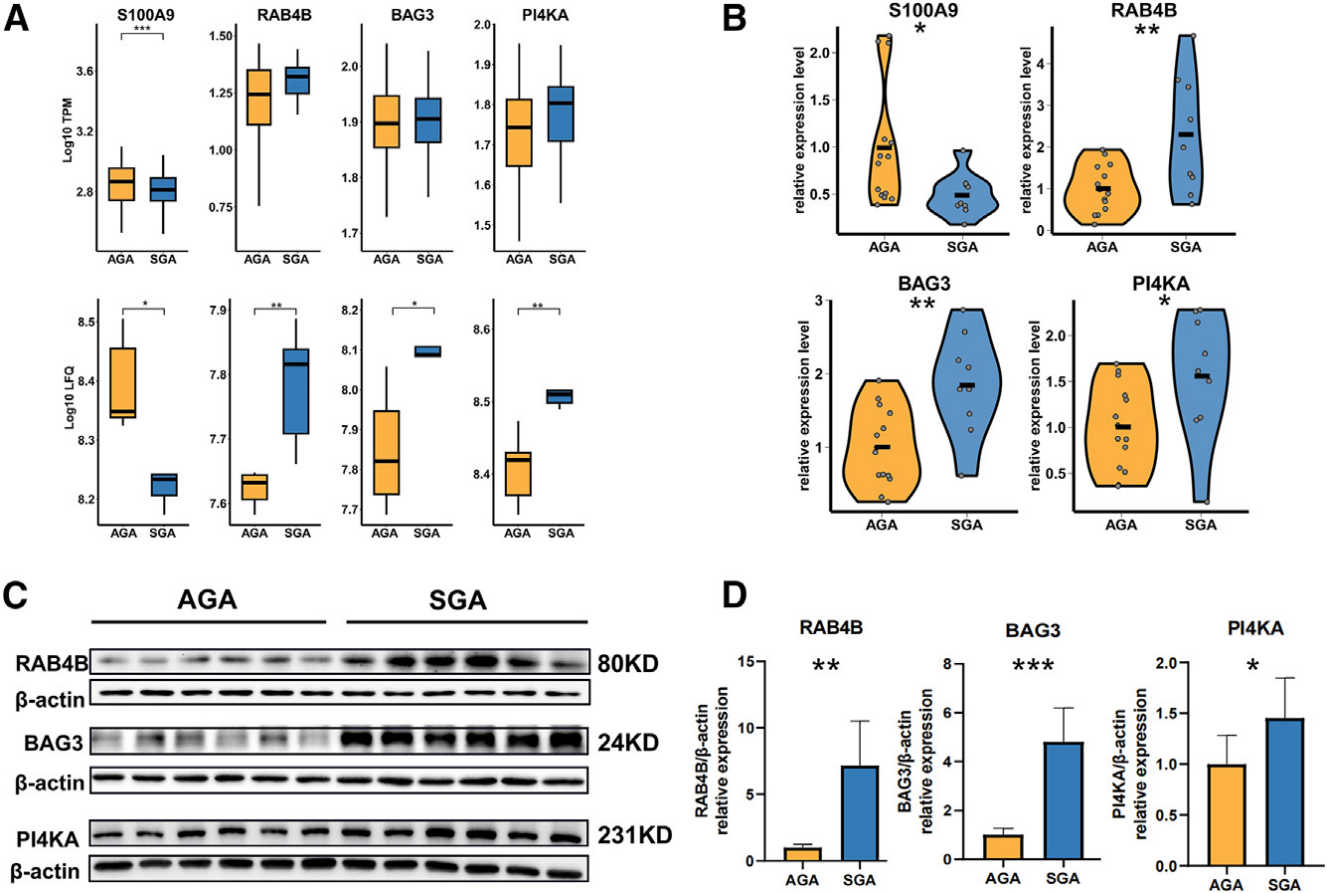
Validation of key regulatory molecules (A) RNA-seq levels and LFQ intensities of four candidate regulatory factors. Boxes represent the IQR with lines showing the median and whiskers denoting the smallest and largest values within 1.5∗IQR. (B) Relative expression levels of regulatory molecules in RT-qPCR. (C) Representative immunoblots of *RAB4B*, *BAG3*, and *PI4KA* proteins in placenta (*n* = 6 per group). (D) Quantification for *RAB4B*, *BAG3*, and *PI4KA*. ∗*p* value<0.05, ∗∗*p* value<0.01, ∗∗∗*p* value<0.001. Data are represented as mean ± SD. The *p* value in RT-qPCR was obtained by Mann-Whitney test, while the *p* value in western blotting was obtained by t test.

Based on the analyses described above, we selected a total of eight key regulatory molecules for further experimental validation. (Figure 7A; Figure S7A). For RT-qPCR validation at RNA level, 23 independent placentas from both the MABC and Chinese National Birth Cohort (CNBC) cohorts were screened, including 14 samples from the AGA group and 9 samples from the SGA group. Based on RT-qPCR data, seven out of eight RNAs showed consistent trend with RNA-seq, of which four RNAs showed significant difference (Figures 7B and S7B). Specially, significantly higher expression of *RAB4B* (*p* value = 0.0253), *BAG3* (*p* value = 0.0097), and *PI4KA* (*p* value = 0.0438) were observed in the placenta from SGA group (Figure 7B). Meanwhile, the expression level of *S100A9* showed significantly decreased in the SGA group (*p* value = 0.0298) (Figure 7B). Moreover, we verified the four genes authenticated by RT-qPCR at the protein level using western blotting. We observed that protein levels of *RAB4B*, *BAG3*, and *PI4KA*, were still significantly increased in the SGA group compared to the AGA group (*p* value = 0.0012, *p* value<0.001, *p* value = 0.045) (Figures 7C and 7D). The protein of *S100A9* failed to be validated in western blotting results. Nevertheless, at both the RNA and protein levels, we ultimately corroborated three key regulatory genes of interest.

## Discussion

In the present study, we generated RNA and protein expression profiles for SGA and AGA using 62 placental tissues at first. Though a systematic analysis of the transcriptomic, we identified DEGs and two SRM networks associated with SGA. Correspondingly, we identified a set of proteins with distinguishable abundance in the SGA placenta compared to controls. The integration of transcriptomic and proteomic data revealed a correlation between mRNA and protein abundance in placental samples, as well as changes in mRNA and protein expression in SGA compared to AGA samples. Finally, we validated the identified core regulatory molecules at both RNA and protein levels through molecular biology experiments such as RT-qPCR and western blot, providing broader insights into the pathological mechanism of SGA.

Processes critical for fetal growth, including placental development, gene expression, hormonal signaling, and inflammation, are typically characterized by sexual dimorphism.^43^ We also identified sex-specific DEGs associated with SGA in human placenta, including those specific to males and females. In particular, consistent with previous findings,^8^ we observed more extensive changes in placental gene expression in female SGA infants. This may be partly attributed to a higher adaptive response in females to mitigate more severe fetal outcomes. Previous research has explored gene expression changes related to SGA using transcriptomic profiling of the human placenta and cord blood. In agreement with the findings by Chatterjee et al.,^8^ we noted that the two aberrant down-regulated RNAs in the SGA group, *ANKRD22*, and *TNFSF13B*. Importantly, the expression levels of these two RNAs were significantly and positively correlated with fetal birth weight. Additionally, imprinted genes, which are critical to fetal growth and development, have been extensively studied.^44^ Our analysis revealed differential expression of the imprinted genes *ERAP2* and *RAC1* between the SGA and AGA groups. *ERAP2*, expressed in the placenta, has been linked to the onset and progression of preeclampsia, while *RAC1* is involved in regulating placental growth.^45^^,^^46^ In the cell type-specific analysis, we observed that both DEGs and DEPs included genes significantly enriched in macrophages. Evidence suggests that an increase in the abundance of these cells is associated with pregnancy complications, including SGA.^47^ In late pregnancy, the increase in macrophage numbers may lead to a reduced fetal growth rate, which could provide a potential explanation.^48^ During placental development, CTB stem cells proliferate and fuse to form the multinucleated STB, which represents the terminally differentiated villous layer and the maternal-fetal interface, where nutrient, gas, and waste exchange between the mother and fetus occurs.^49^ Specifically, the STB layer is at the forefront of nutrient exchange and also performs essential endocrine functions crucial for maintaining pregnancy and fetal development, while appropriate regulation of CTB differentiation and invasion is considered vital for a successful pregnancy.^49^^,^^50^ Given that proteins carry out most cellular functions, we also observed that CTB is the cell type with the highest enrichment of DEPs. This suggests that the regulatory role of proteins in CTB within the placenta of SGA infants warrants further investigation. We also observed that EVT and STB cells are, respectively, enriched in DEPs and SRM (yellow module) genes. These features further reveal that trophoblast cell invasion may be a potential factor in the mechanisms underlying SGA.^51^ These findings in the proteome also suggest that future research should focus on the role of potential post-translational modifications in the mechanism of SGA. The previous consistency partly indicated the reliability of our dataset, and also provided richer resources for the mechanism study of SGA.

In the study of the functional pathways of various regulatory molecules, the DEGs were centrally enriched for immune-related terms, highlighting the central role of this canonical pathway. Surely, the immune system is a key link in pregnancy and fetal development.^52^ Importantly, the regulatory role of certain immune genes in the placenta of SGA infants have also been recognized, further highlighting the role of the immune system in SGA.^9^^,^^53^^,^^54^ Another important pathway involves receptor binding and signaling, indicating that the mechanisms underlying SGA could be further understood through these processes. In addition, we found that the dysregulated proteins may be related to the multiple cellular processes and extracellular organizations, which mean that intercellular communication may be associated with SGA, while previous studies have also provided some clues in this regard. For example, genetic variants in extracellular matrix-related genes were observed to show a significant association with SGA in a case-control study, and elevated concentrations of circulating fibronectin were concentrated with an increased risk of SGA.^55^ Notably, metabolic pathways were enriched in DEPs and SRM genes, regulating at both the protein and RNA levels. Extensive studies mentioned that SGA as posing a elevated future risk of metabolic disorders and metabolic diseases.^56^^,^^57^ Additionally, SGA status has been reported to have a lasting impact on neurodevelopmental outcomes.^58^ Our study identified DEPs that are enriched in the amyotrophic lateral sclerosis pathway. Consequently, we conclude that the diverse molecular signatures observed in the SGA placenta may either drive placental abnormalities or reflect enduring effects on the offspring’s health.

Next, we inquired about the relationship between mRNA and protein levels in the placenta. We revealed a significant correlation, implying that this finding could broaden the exploration horizon of the mRNA-protein relationship. Meanwhile, we investigated whether the log2 FC of genes remains relevant at the mRNA and protein levels in the comparison of SGA with AGA. In a preceding transcriptomic and proteomic analysis of placental tissue with placenta accreta, the log2 FC of genes displayed a weak correlation at mRNA and protein levels in comparison of placenta accreta to control (R = 0.033).^59^ In contrast, the correlation derived in SGA compared to AGA is considerably stronger (R = 0.16), which is also an interesting finding that we have proposed. Importantly, we characterized a panel of genes that varied coherently at the mRNA and protein levels, which were significantly enriched in the haptoglobin binding, hemoglobin complex, and oxygen carrier activity terms. Indeed, the placenta governs the transport of oxygen and nutrients between the mother and fetus, suggesting that dysregulation of genes in the “Consistent” panel triggers the incidence of SGA by impairing placental oxygen transport processes. Haptoglobin, an acute-phase protein with hemoglobin-binding capabilities, is involved in immune responses and inflammatory processes.^60^ Previous studies have linked maternal hemoglobin concentration in late pregnancy to the risk of low birth weight and SGA.^61^ We suggest that placental haptoglobin might contribute to the pathophysiology of SGA by modulating immune responses and inflammatory processes or by interfering with placental function through hemoglobin binding, potentially affecting fetal growth. These findings contributed clues to decode the mechanism of SGA. A prior study in mammalian fibroblast cell populations calculated that approximately 40% of the variance at the protein level could be explained by mRNA levels,^62^ consistent with findings in human brain tissues.^25^ We noticed that the correlation value obtained in our study was weaker compared to them. One possible explanation could be that the formation of the human maternal-fetal interface involves sophisticated spatial and temporal coordination, and the placenta, as a complex heterogeneous organ, comprises cells of maternal and fetal origin that are inextricably coupled.^63^^,^^64^ Furthermore, Wilhelm et al.^65^ reported mRNA-protein expression correlations across 12 human tissues. Our findings reveal that the mRNA-protein correlation in placental tissue mirrors this pattern, remaining at similarly low levels. This comparison highlights the generally weak correlation between protein and mRNA levels, implying a broad implementation of regulatory mechanisms affecting transcription, translation, and post-translational control of protein abundance. That being said, we still captured the significance feature because of the *p* value less than 0.001. As previously mentioned, in mRNA-protein correlation analyses involving over a thousand data points, even a low R^2^ value can show high significance (e.g., *p* value <0.001). This underscores the importance of considering both correlation coefficients and *p* values when evaluating the overall correlation features of the dataset.^25^

Finally, we assessed the relative abundance of core candidate molecular signatures associated with SGA using RT-qPCR and western blotting. Three candidate causal genes (*RAB4B*, *BAG3*, and *PI4KA*) in the SGA group were significantly distinguished from the AGA group at both the RNA and the protein levels. To date, there is no detailed description of *RAB4B*’s role in the placenta. Previous studies have indicated that *RAB4B*, a protein involved in endocytic recycling, is regulated by MHC class II genes, suggesting its potential involvement in antigen presentation and highlighting its importance in the immune system.^66^ We hypothesize that abnormal *RAB4B* expression might offer insights into immune system disruption during placental development. *BAG3*, a multifunctional protein that links various signaling pathways,^67^ was found to be associated with several enriched signaling pathways in our DEGs. Therefore, we proposed that the mechanism of *BAG3* regulation in the SGA is related to its function in signal transduction. Additionally, *PI4KA* represents one of the important phosphatidylinositol kinases involved in complex human diseases, a regulatory mediator in developmental disorders,^68^^,^^69^ which was also considered as a key molecular feature involved in fetal growth and development in our study. Regretfully, *S100A9* was validated in RT-qPCR, whereas not detected at the protein level by western blotting. This discrepancy may be due to the low expression levels of *S100A9* in the placenta and potential protein degradation in the samples.^70^

To the best of our knowledge, this is the specific study that integrating transcriptome and proteome profiling to characterize the molecular features of the placenta in SGA infants. By combining both transcriptomic and proteomic analyses, our study offers a more comprehensive view of the biological and pathological mechanisms underlying SGA, with a direct connection to the disease phenotype. Meanwhile, we have systematically explored the discrepancy and relevance of mRNA and protein expression in the placenta to provide complementary resources for understanding the hierarchy of gene expression pathways. Another important strength is that we have validated three core regulatory genes associated with SGA in the independent cohort, both at RNA and protein level, increasing the credibility of the findings.

Taken together, this study comprehensively analyzed human placental transcriptomic and proteomic profiles, describing molecular features associated with SGA and key pathways with potential synergistic regulatory effects. These findings provide a foundation for further investigation of the biological mechanisms of SGA, and the identification of pivotal regulatory genes that underpin the pathomechanism of SGA may reveal therapeutic targets to boost fetal growth and prognosis health.

### Limitations of the study

Our work has limitations. First, while it is encouraging that the key regulatory genes we identified did not overlap with previous findings, the limited number of validation samples and the extended freezing of placental tissue may have impacted the experimental results. Second, given the study only based on a single-center population of Chinese people, further validation of inferences in large multi-ethnic cohorts is warranted in the future. Last, since fetal growth and development are influenced by genetic factors, future research should incorporate additional genomic data to better elucidate the regulatory mechanisms involved.

## Resource availability

### Lead contact

Further information and requests for resources and reagents should be directed to the lead contact, Yumin Zhu (zhuyumin@nju.edu.cn).

### Materials availability

This study did not generate new unique reagents.

### Data and code availability

• Data: The RNA sequencing datasets in the current study were submitted to the NCBI Sequence Read Archive (SRA) under study accession number PRJNA1027796 and PRJNA906459. The mass spectrometry proteomics datasets have been deposited to the ProteomeXchange Consortium (http://proteomecentral.proteomexchange.org) via the iProX partner repository^71^ with the dataset identifier PXD046307 and PXD046308.
• Code: This paper does not report original code.
• Additional information: Any additional information required to reanalyze the data reported in this paper is available from the lead contact upon request.

## Acknowledgments

We thank all the participants involved in the study. We are grateful to the Scientific Research Center in Preventive Medicine, School of Public Health, Anhui Medical University for the technical support in our experiment. This work was supported by the 10.13039/501100001809National Natural Science Foundation of China (82103870), the 10.13039/501100012226Fundamental Research Funds for the Central Universities (14380541), Key Project of Translational Medicine Research Institute of Anhui Province (2022zhyx-B06), Postgraduate Innovation Research and Practice Program of Anhui Medical University (YJS20230050), and 10.13039/501100003995Natural Science Foundation of Anhui Province (2108085QH360).

## Author contributions

Conceptualization, H.J. and Y.Z.; methodology, X.W., L.L., C.R., and Y.Z.; formal analysis, H.J.; Data curation, H.J. and Y.Z.; visualization, H.J.; investigation, H.J., X.W., C.R., and L.L.; validation, H.J., X.W., and L.L.; writing-original draft, H.J.; review and editing, Y.Z.; resources, H.G., Q.W., F.T., and Y.Z.; supervision, Q.W., F.T., and Y.Z.; project administration, Q.W., F.T., and Y.Z.

## Declaration of interests

The authors declare no competing interests.

## STAR★Methods

### Key resources table

**Table:** 

REAGENT or RESOURCE	SOURCE	IDENTIFIER
**Antibodies**		

Mouse monoclonal anti-Rab4B antibody	Santa Cruz Biotechnology	Cat# sc-271982; RRID: AB_10709179
Rabbit polyclonal anti-Pi4ka antibody	Fine Test	Cat# PAB3227; RRID: AB_1578723
Rabbit monoclonal anti-Bag3 antibody	Abcam	Cat# ab92309; RRID: AB_2049196

**Biological samples**		

Human placental tissue	This study	N/A

**Critical commercial assays**		

Reverse transcription reagent	ACCURATE BIOTECHNOLOGY	A4A2934
qPCR SYBR Green Master Mix	YESEN	Cat# 11201ES08

**Deposited data**		

RNA-sequencing data	This paper	Sequence Read Archive:PRJNA1027796, PRJNA906459
Proteome data	This paper	iProX:PXD046307, PXD046308

**Oligonucleotides**		

Primers for RT-qPCR, see Table S2	This paper	N/A

**Software and algorithms**		

Cytoscape [v3.10.1]	Shannon et al.^72^	RRID:SCR_003032
edgeR [v3.40.2]	Robinson et al.^73^	RRID: SCR_012802
HISAT2 [v2.2.0]	Kim et al.^74^	RRID: SCR_015530
HOMER [v4.11.0]	http://homer.ucsd.edu/homer/	RRID: SCR_010881
MaxQuant [v1.6.2]	Cox et al.^75^	RRID:SCR_014485
R [v4.2.3]	https://www.R-project.org/.	RRID:SCR_001095

### Experimental model and study participant details

#### Sample collection

All placental tissue samples from pregnant women were delivered between 2013 and 2015 from MABC cohort. Only singleton live births and samples with full-term were considered for selection. After matching fetal sex, maternal ages, and gestational weeks, we collected 62 samples including 31 SGA samples referring to the definition, and 31 AGA samples as control.^76^ Additionally, a total of 23 placental samples (SGA *n* = 9, AGA *n* = 14) were integrated using consistent selection criteria to form an independent cohort for validation experiments. Of these, 14 samples were derived from the MABC cohort, while the remaining 9 were obtained from the CNBC cohort. Informed consent was obtained from all subjects. The CNBC cohort is a prospective birth cohort conducted in Ma’ anshan City, Anhui Province, China, recruited between May 2017 and September 2018, and included a group of pregnant women aged 18 years or older who conceived naturally. Ethical approval for the study was obtained from the Ethics Committee of Anhui Medical University (No. 20131195).

Placental tissue was sampled within 30 min of delivery. A piece of the intact placental leaflet was removed at 5 cm from the umbilical cord and sampled to a depth of about 1–2 cm, avoiding the fascia, washed with saline, and then cut longitudinally into tissues smaller than 0.5 cm. The harvested placental leaflets were cut longitudinally into four equal-sized slices, each containing the fetal and maternal sides. The tissue was completely immersed in RNA later and refrigerated overnight at 4°C, then transferred to lyophilized tubes for long-term storage at −80°C when the supernatant was poured off.

### Method details

#### High-throughput transcriptomics and proteomics

To produce RNA libraries, 1μg total RNA extracted from each sample was blended with magnetic beads with Oligo (dT) to isolate RNAs with poly-A tails after fragmentation of mRNAs. After the synthesis of double-stranded cDNA, purification was carried out using AMPure XP beads, followed by paired-end sequencing using Illumina NovaSeq 6000. To ensure the quality of subsequent analyses, we removed adapter sequences and filtered low-quality (the number of bases with base quality values less than or equal to 25 accounted for more than 60% of the entire reads) and N (no base information could be determined) reads greater than 5%. Quality data for each sample can be accessed in Table S4. The acquired clean reads were sequence aligned with the human reference genome hg38 using HISAT2 software, and the expression of each gene in each sample was calculated by the featureCounts.^74^ The count data were transformed to TPM for going normalization by the “edgeR” package in the R platform.^73^

Label-free proteomic analysis was also applied to SGA and AGA placental tissues which were also employed for RNA sequencing. The proteome was quantified by LC-MS assay using sample combining, and each group was combined into six samples for the assay. The extracted proteins were digested with trypsin using Filter aided proteomic preparation (FASP), and then obtain MS data underlying LC-MS measurements.^77^ Moreover, the obtained MS data were analyzed with the software MaxQuant and quantified based on the label-free quantification (LFQ) algorithm.^75^^,^^77^^,^^78^ For LC-MS, we dissolved peptides in buffer solution A (0.1% formic acid) and buffer solution B (0.1% formic acid and 84% acetonitrile) and used the HPLC liquid phase system Easy nLC to separate the samples. The samples were separated by chromatography and analyzed via mass spectral on a Q-Exactive mass spectrometer. The detection mode was positive ion, and the scanning range of the parent ion was 300∼1800 m/z. The primary mass resolution was 70,000 at 200 m/z. The detection mode was positive ion, the scanning range of the parent ion was 300∼1800 m/z, the resolution of the primary mass spectral was 70,000 at 200 m/z. We set the Automatic gain control (AGC) target to 1e6, the Maximum IT to 50ms, and the dynamic exclusion time to 60s. The variable modifications were oxidation, while the fixed modification was carbamidomethylation of cysteine. Main search was set to 6 ppm, while first search and MS/MS tolerance were both set to 20 ppm. To obtain high-quality MS experimental data, we analyzed the MS spectral data using the rigorous analysis tool Andromeda and adopted protein FDR≤0.01 as the screening criterion. The data regarding protein quantities before and after filtering are provided in Table S5.

DEGs were configured as absolute |log2 FC| ≥ 0.263, FDR <0.1, calculated by edgeR.^73^ T test was used to assess the difference in LFQ intensity of proteins between the SGA and AGA group. Meanwhile, the yield of *p*-value <0.05 and absolute |log2 FC| ≥ 0.263 was consulted for DEPs.

#### Gene co-expression network analysis

The analysis of WGCNA motivated by all genes obtained after filtering with an average expression value >1, was compiled for discovering modules containing a cluster of highly related genes.^79^ The main parameters were as follows: Power = 8, TomType = unsigned, minModuleSize = 30, mergeCutHeight = 0.25, reassignTreshold = 0, and the remaining default parameters. We computed the Pearson correlation coefficient between the modules and traits to select modules with the most significant and strongest correlations for further analysis. Then, we evaluated the preservation of the module via the modulePreservation function, specifically characterized by the Z-summary score (*Z* score). In general, it is considered that a *Z* score greater than 10 as a judgment criterion.^80^^,^^81^ Visualization of the gene networks within the key modules was carried out using Cytoscape and NetworkAnalyst, with gene connectivity measured by Maximal Clique Centrality (MCC) in Cytoscape.^72^^,^^82^^,^^83^

#### RT-qPCR

Total RNA was executed using the Trizol reagent (Invitrogen), and converted to cDNA with Evo M-MLV reverse transcription reagent premix (ACCURATE BIOTECHNOLOGY). Complete the configuration of the amplification system by incorporating the premixed solution SYBR green (YESEN) into the cDNA template for subsequent amplification in a LightCycler 96 System (Roche), following the run procedures provided by instructions. Glyceraldehyde-3-phosphate dehydrogenase (GAPDH) was used as a reference housekeeping gene in comparisons of gene expression.^84^ The primer sequences are provided in the supplementary information (Table S2). The amplification procedure was performed for a total of 40 cycles, and the gene was considered undetectable when the CT value exceeded 35.^85^ We used the 2-ΔCT method to normalize the data to obtain the relative expression levels of each gene.

#### Western blotting

Total proteins in the placenta were withdrawn with RIPA buffer. Protein concentrations were measured complying with the company’s instructions using the Pierce BCA Protein Analysis Kit (Thermo Scientific). Proteins were isolated at 10–30 μg in 10%–15% SDS-PAGE after denaturing, and the SDS-PAGE proteins later were placed on polyvinylidene fluoride (PVDF) membranes. PVDF membranes were demonstrated in a solution of 5% skim milk. After that, primary antibodies were applied to indicate the PVDF membranes for 1–3 h. The PVDF membrane was washed and then incubated with the matching secondary antibody for 1–2 h. In the end, the signals were assayed through the application of a highly sensitive chemiluminescent reagent. The quantitation of the target proteins was conducted in Image Pro Plus software.

#### Annotation gene sets

The list of disease-related genes was obtained from the UniProt database.^86^ Cancer-related genes and drug targets genes were reaped from the open access resources provided by HPA project.^23^ GPCRs genes were integrated from the International Union of Basic and Clinical Pharmacology (IUPHAR) and HUGO Gene Nomenclature Committee (HGNC) database.^87^^,^^88^ The collections of mitochondrial genes and phosphatase genes were derived from MitoCarta 3.0 and Dephosphorylation Database (DEPOD), respectively.^89^^,^^90^ The list of TF genes was downloaded from the HumanTFs.^91^ From a 2019 study by Pique-Regi et al.,^64^ a 2018 study by Vento-Tormo et al.,^92^ and a 2018 study by Liu et al.,^93^ we collected cell-specific gene sets to assess the cellular specificity of the genes of interest. We culled gene lists of SGA-associated diseases by searching in GeneCards and filtered several genes based on the median of the provided match scores (Table S6).^94^ The formation of imprinted gene lists from the following resources: Geneimprint, Catalog of Parent of Origin Effects, and two studies about placental imprinted gene expression.^37^^,^^38^^,^^39^^,^^40^ Resources on evidence and classification of proteins, all previously described, are available from HPA, UniProt, and neXtProt consortia.^23^^,^^86^^,^^95^

#### Pathway enrichment analysis

Pathway analyses were performed using g: Gost supported by g:Profiler,^96^ which can detect statistically significantly enriched terms of Gene Ontology (GO) and KEGG pathways. The top pathways are filtered by adjusted *p*-value.

#### TF analysis

To locate TFs upstream of key regulatory genes, we performed the TF motif analysis using Hypergeometric Optimization of Motif EnRichment (Homer) function “findMotifs.pl”.^97^

### Quantification and statistical analysis

For the assessment of the enrichment of DEGs and DEPs in the set of genes of interest, we carried out a two-sided Fisher’s exact test at a *p*-value threshold of 0.05 in R v4.2.3 software, also known as over-representation analysis (ORA). For correlation analysis, we used the Spearman rank correlation test implemented through the “cor. Test” function in R, since our data fall out of the strict normal distribution. The results obtained by RT-qPCR were analyzed using the Mann-Whitney test without normal distribution and western blotting results are consistent with normal distribution and were calculated using the T test. All significant differences in statistical tests were considered *p*-value <0.05. All figures were created using R v4.2.3 software (https://www.R-project.org/).

## Supplemental information

Document S1. Figures S1–S7 and Tables S1–S3

Table S4. Sample quality data for the transcriptome

Table S5. Quality control data for the proteome

Table S6. Disease-associated gene lists present in different gene sets
