# Supplementary material for: Integrated proteomic and transcriptomic landscape of human placenta in small for gestational age infants
Source: iScience. 2024 Nov 19;27(12):111423. doi: 10.1016/j.isci.2024.111423 (PMC11648249; doi:10.1016/j.isci.2024.111423)
Supplement: Document S1. Figures S1–S7 and Tables S1–S3 [file mmc1.pdf]

iScience, Volume *27*

Supplemental information

Integrated proteomic and transcriptomic landscape of human placenta in small for gestational age infants

Heyue Jin, Xianyan Wang, Lingyu Li, Chen Rui, Hong Gan, Qunan Wang, Fangbiao Tao, and Yumin Zhu

Supplemental information

Supplementary figures and tables

Table S1 Characteristics for the 31 pairs of SGA cases and controls in RNA-sequencing

Characteristics	SGA (n=31)	AGA (n=31)
Maternal age (years), mean (SD)	27.7 (2.8)	27.8 (3.0)
Maternal BMI (kg/m^2), mean (SD)	19.6 (2.2)	20.0 (2.1)
Parity, n (%)		
Multipara	3 (9.7)	3 (9.7)
Nulliparous	28 (90.3)	28 (90.3)
GDM, n (%)		
Yes	2 (6.5)	3 (9.7)
No	29 (93.5)	28 (90.3)
PPROM, n (%)		
Yes	5 (16.1)	6 (19.4)
No	26 (83.9)	25 (80.6)
Placental previa		
Yes	0 (0.0)	1 (3.2)
No	31 (100.0)	30 (96.8)
Mode of delivery, n (%)		
Cesarean section	13 (41.9)	12 (38.7)
Vaginal delivery	18 (58.1)	19 (61.3)
Sex, n (%)		
Female	15 (48.4)	16 (51.6)
Male	16 (51.6)	15 (48.4)
Gestational weeks, mean (SD)	39.6 (0.5)	39.5 (0.5)
Birth weight (g), mean (SD)	2807.1 (126.8)	3432.9 (265.9)

SGA: small for gestational age; AGA: average for gestational age; GDM: Gestational diabetes mellitus; PPROM: preterm premature rupture of membranes.

Table S2 Gene primer sequences used in RT-qPCR.

Gene	Forward Primer	Reverse Primer
RAB4B	ATGGCTGAGACCTACGACTTC	CGCCGATTGTGTGGTTGGA
BAG3	TGGGAGATCAAGATCGACCC	GGGCCATTGGCAGAGGATG
PI4KA	CAGCTCTGACCAAGTGGAGAT	GCGGATGGTTGCATTTGGAA
S100A9	ACATCATGGAGGACCTGGACAC	AGGTTAGCCTCGCCATCAGC
TMEM40	CAGAGCAACCGGAAAACATCG	TCATCCTTCAAAACGTCAGGC
HP1BP3	CCATGCCGATTCGTCGAACT	CCTCACTCGAAGTAGCAGGT
LAMB2	CTCAGCCCAGCTCCTGGGGAGAAGG	GAGTCGGGTCGAGGACCCCTCTTCC
NUP205	TTTGGCGGTAAATTCGGCTG	AGAAGTCGAGTTCCCTGTTGA
GAPDH	GAACGGGAAGCTTGTCATCAA	ATCGCCCCACTTGATTTTGG

Table S3 Information of the enriched TF-binding motifs.

Motif_Name*	TF	p-value
DUX(Homeobox)/C2C12-Dux-ChIP-Seq(GSE87279)/Homer	DUX	0.001
PU.1-IRF(ETS:IRF)/Bcell-PU.1-ChIP-Seq(GSE21512)/Homer	SPI1	0.001
IRF4(IRF)/GM12878-IRF4-ChIP-Seq(GSE32465)/Homer	IRF4	0.001
EWS:ERG-fusion(ETS)/CADO_ES1-EWS:ERG-ChIP-Seq(SRA014231)/Homer	EWG	0.001
Ascl2(bHLH)/ESC-Ascl2-ChIP-Seq(GSE97712)/Homer	ASCL2	0.001
ZFX(Zf)/mES-Zfx-ChIP-Seq(GSE11431)/Homer	ZFX	0.001
Npas4(bHLH)/Neuron-Npas4-ChIP-Seq(GSE127793)/Homer	NPAS4	0.001
Cux2(Homeobox)/Liver-Cux2-ChIP-Seq(GSE35985)/Homer	CUX2	0.001
RUNX2(Runt)/PCa-RUNX2-ChIP-Seq(GSE33889)/Homer	RUNX2	0.001

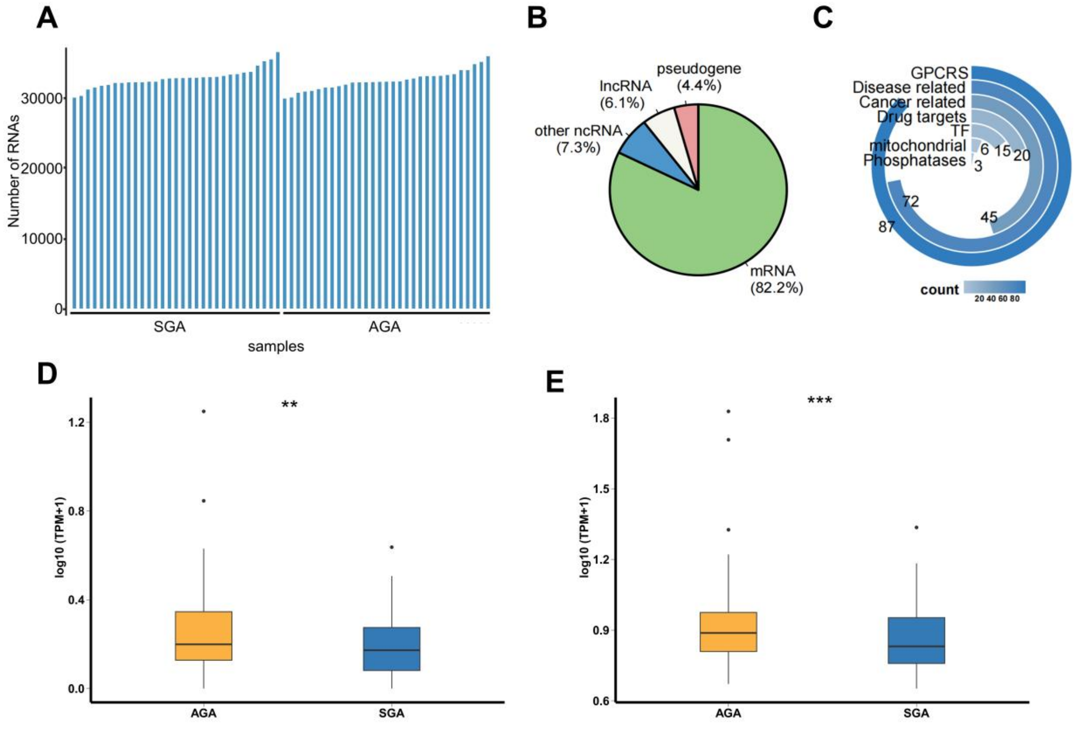

Figure S1 The features of transcriptomic profiles.

(A) The number of RNA detected per sample. Each bar represents a sample.

(B) Types of differentially expressed RNAs.

(C) Distribution of DEGs in selected functional categories.

(D) Boxplot of *ANKRD22* expression levels between the AGA and SGA groups.

(E) Boxplot of *TNFSF13B* expression levels between the AGA and SGA groups. Boxes represent the IQR with lines showing the median and whiskers denoting the smallest and largest values within 1.5*IQR. **p-value<0.01, ***p-value<0.001. The p-value was obtained by Mann-Whitney test.

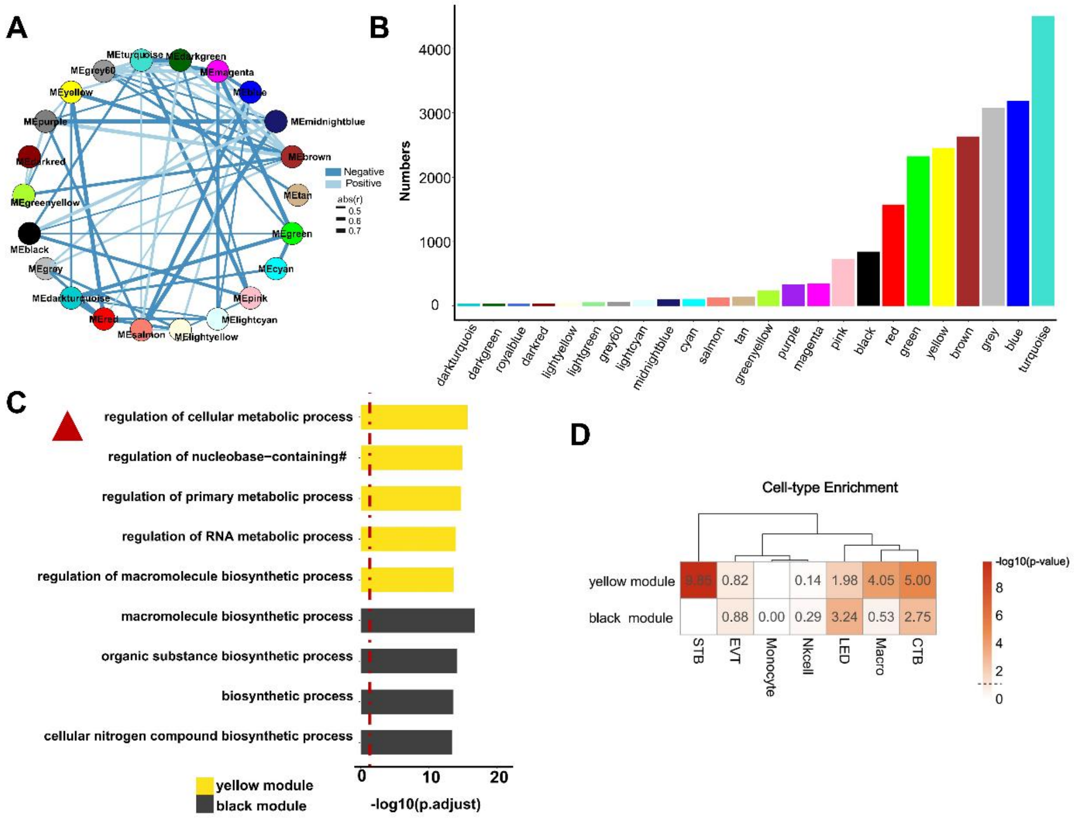

Figure S2 Analysis of SGA-related module networks.

(A) The network diagram demonstrates the relationship between modules. The weights of the edges are determined by the strength of the association between eigengenes of modules. Here, only the pairs of relationships showing p-value < 0.001 are retained for display.

(B) Numbers of genes in each modules.

(C) Enrichment of the BP term in SRMs. The red triangles mark the shared terms between the two modules. “#” means that the name is abbreviated, and the full name is “regulation of nucleobase-containing compound metabolic process”. The red dotted line represents adjusted p-value < 0.05.

(D) Cellular specificity of SRMs.

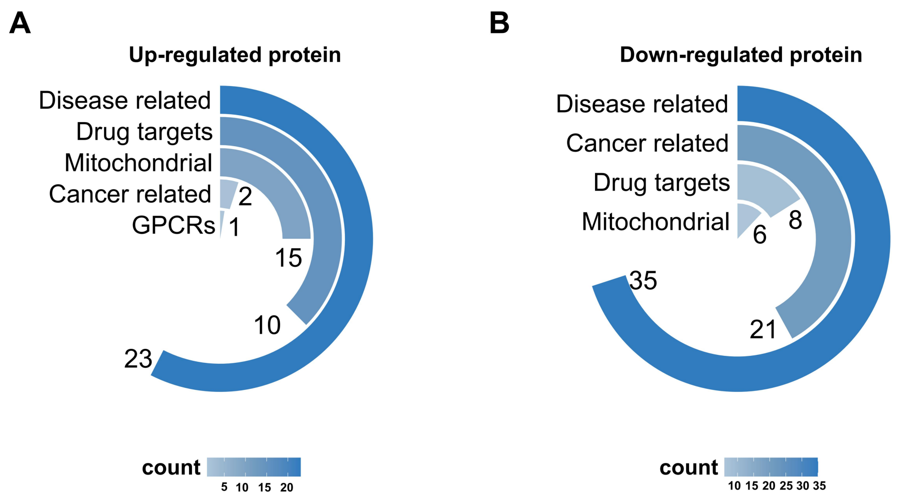

Figure S3 Distribution of DEPs functions.

(A) Distribution of up-regulated proteins in selected functional categories.

(B) Distribution of down-regulated proteins in selected functional categories.

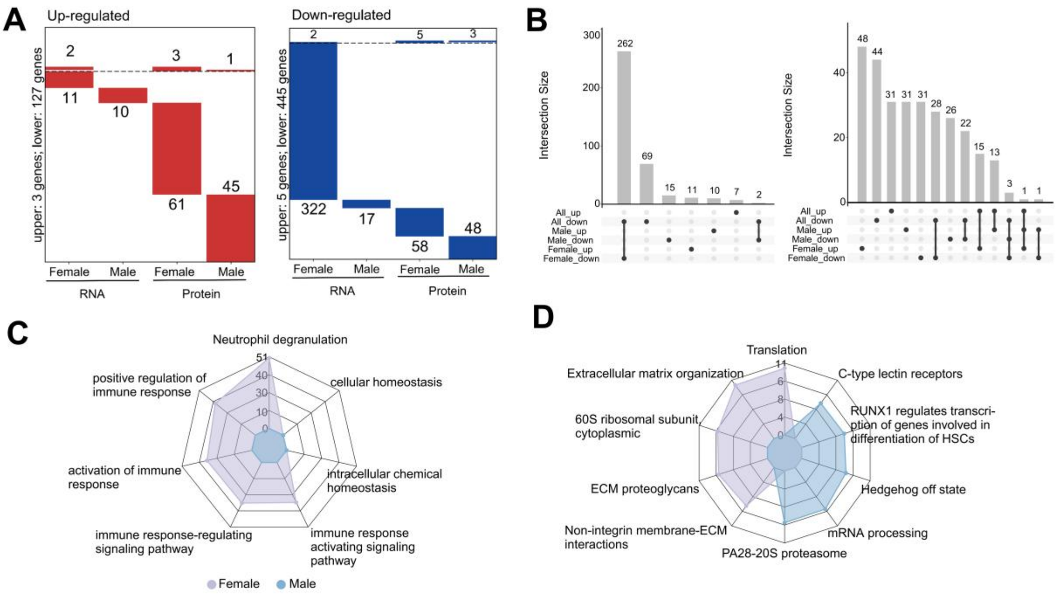

Figure S4 Sex-specific signatures of SGA placenta.

(A) The distribution of DEGs and DEPs for male and female in placenta between the SGA and AGA. Red: up-regulated. Blue: down-regulated. The upper part (above the dotted line) indicates DEGs or DEPs that overlap in at least two groups, and the lower part (below the dotted line) indicates DEGs or DEPs that are unique in each group.

(B) Comparison of DEGs and DEPs identified in sex-specific analyses with those obtained from sex-independent analyses. Left: DEGs. Right: DEPs. All: not grouped by fetal sex.

(C) Radar plot showing pathway enrichment of sex-specific DEGs. Numbers on the axes indicate -log10 adjusted p-value. Only the top5 sex-specific pathways are shown.

(D) Radar plot showing pathway enrichment of sex-specific DEPs.

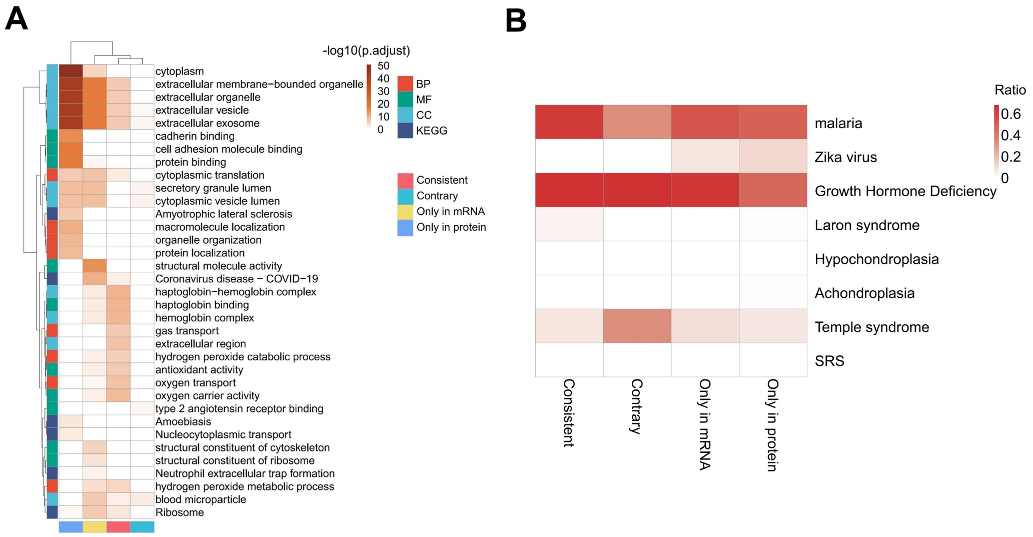

Figure S5 Potential roles of mRNAs and proteins in different expression patterns.

(A) The top 3 GO-terms and KEGG pathways enriched in the gene sets with different expression patterns.

(B) Proportion of disease-associated gene lists present in different gene sets. SRS, Silver–Russell syndrome.

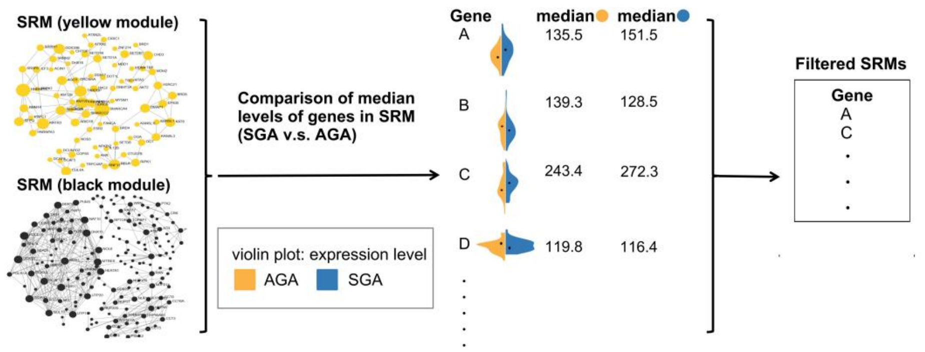

Figure S6 Filters for genes in the SRMs.

Filters for genes in the SRMs. For each gene in the SRMs (yellow module and black module), its median level in the SGA and AGA groups was compared. The violin plot displayed the expression level of each gene in the SRMs and the black point in the violin represents the median value. For example, genes *B* and *D* were factored out in the final filtered SRMs because their median level in the SGA was lower than AGA group.

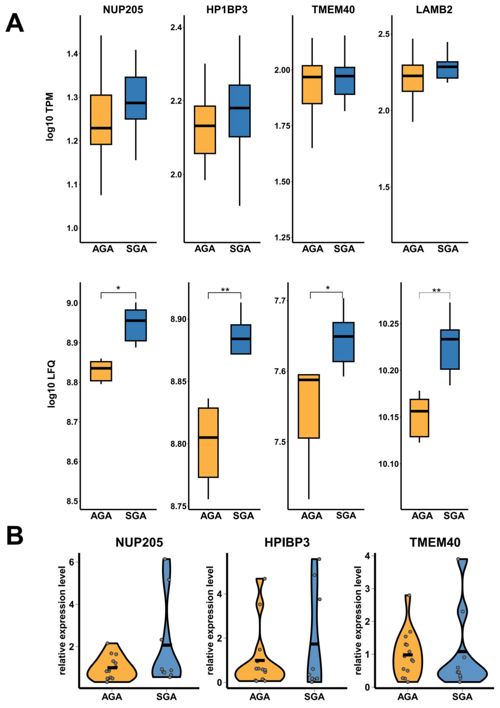

Figure S7 The expression levels of other candidate regulatory molecules.

(A) RNA-seq levels and LFQ intensities of other four candidate regulatory factors. Boxes represent the IQR with lines showing the median and whiskers denoting the smallest and largest values within 1.5*IQR.

(B) Relative expression levels of *NUP205*, *HP1BP3*, and *TMEM40* in RT-qPCR. *p-value < 0.05, **p-value < 0.01, ***p-value < 0.001.
